# Genetic and Epigenetic Intersections in COVID-19-Associated Cardiovascular Disease: Emerging Insights and Future Directions

**DOI:** 10.3390/biomedicines13020485

**Published:** 2025-02-16

**Authors:** Hussein Sabit, Borros Arneth, Afaf Altrawy, Aysha Ghazy, Rawan M. Abdelazeem, Amro Adel, Shaimaa Abdel-Ghany, Amany I. Alqosaibi, Panos Deloukas, Zulfugar T. Taghiyev

**Affiliations:** 1 Department of Medical Biotechnology, College of Biotechnology, Misr University for Science and Technology, Giza P.O. Box 77, Egypt; 2 Institute of Laboratory Medicine and Pathobiochemistry, Molecular Diagnostics, Hospital of the Universities of Giessen and Marburg (UKGM), Justus Liebig University Giessen, 35392 Giessen, Germany; 3 Department of Agri-Biotechnology, College of Biotechnology, Misr University for Science and Technology, Giza P.O. Box 77, Egypt; 4 Department of Environmental Biotechnology, College of Biotechnology, Misr University for Science and Technology, Giza P.O. Box 77, Egypt; 5 Department of Biology, College of Science, Imam Abdulrahman bin Faisal University, Dammam 31441, Saudi Arabia; 6 William Harvey Research Institute, Barts and the London School of Medicine and Dentistry, Queen Mary University of London, London E1 4NS, UK; p.deloukas@qmul.ac.uk; 7 Department of Cardiovascular Surgery, Hospital of the Universities of Giessen and Marburg (UKGM), Justus Liebig University Giessen, 35392 Giessen, Germany

**Keywords:** COVID-19, cardiovascular disease, miRNA biomarkers, ticagrelor resistance, clopidogrel efficacy, endothelial dysfunction

## Abstract

The intersection of COVID-19 and cardiovascular disease (CVD) has emerged as a significant area of research, particularly in understanding the impact of antiplatelet therapies like ticagrelor and clopidogrel. COVID-19 has been associated with acute cardiovascular complications, including myocardial infarction, thrombosis, and heart failure, exacerbated by the virus’s ability to trigger widespread inflammation and endothelial dysfunction. MicroRNAs (miRNAs) play a critical role in regulating these processes by modulating the gene expressions involved in platelet function, inflammation, and vascular homeostasis. This study explores the potential of miRNAs such as miR-223 and miR-126 as biomarkers for predicting resistance or responsiveness to antiplatelet therapies in COVID-19 patients with cardiovascular disease. Identifying miRNA signatures linked to drug efficacy could optimize treatment strategies for patients at high risk of thrombotic events during COVID-19 infection. Moreover, understanding miRNA-mediated pathways offers new insights into how SARS-CoV-2 exacerbates CVD, particularly through mechanisms like cytokine storms and endothelial damage. The findings of this research could lead to personalized therapeutic approaches, improving patient outcomes and reducing mortality in COVID-19-associated cardiovascular events. With global implications, this study addresses the urgent need for effective management of CVD in the context of COVID-19, focusing on the integration of molecular biomarkers to enhance the precision of antiplatelet therapy.

## 1. Introduction

COVID-19-associated acute cardiovascular syndrome comprises a wide range of clinical manifestations such as abrupt heart damage, cardiomyopathy, and hemodynamic instability. Of COVID-19 patients in China, 7–33% experienced myocardial damage, arrhythmias, cardiac arrests, heart failure, and coagulation abnormalities [1]. The SARS-CoV-2 S protein can directly invade cells because it can attach to human ACE2. At the S1/S2 cleavage site of SARS-CoV-2, you may find the polybasic insertion (PRRAR), which Furin is capable of cleaving. It seems that this new cleavage site helps SARS-CoV-2 cross the S1/S2 barrier and digest the S protein, which is required for cellular entry. The viruses that cause avian flu, which may be fatal, and Newcastle disease, have similar cleavage sites. This unique feature is thought to play a significant role in the multicellular tropism of SARS-CoV-2, which amplifies the multiorgan effects of COVID-19 [2].

SARS-CoV-2 infection causes myocardial harm through a variety of pathways, both direct and indirect. The most largely acknowledged mechanisms include direct viral invasion, dysregulated immunological responses, hypoxemia caused by respiratory failure, and thrombosis-induced infarction. A thorough knowledge of the mechanism of cardiac injury is critical for early detection and illness diagnosis as well as the creation of more effective therapies [3].

Widespread endothelial inflammation has been related to direct SARS-CoV-2 infection of endothelial cells [4]. Inflammation and cytokine storms, including endothelial cell and macrophage activation, are present in individuals with severe COVID-19. Interleukin-1, IL-6, IL-8, and TNF-α levels are also elevated. Despite taking preventative anticoagulants, a group of COVID-19 patients at high risk of venous thromboembolism (VTE) had a hypercoagulable state, according to emerging research [5]. 

Virus invasion of the vascular endothelium and myocardium may cause direct injury to cardiac cells, which might explain the cardiovascular harm linked to COVID-19 [6]. Another theory holds that the systemic inflammation brought on by cytokine storm induces tissue hypoxia, coronary plaque instability, and micro-thrombogenesis [6]. Additionally, it has been determined that a genetic propensity to cardiac events connected to COVID-19 may play a role in the higher death rate among African American COVID-19 patients [7]. It is critical to investigate the genetic composition and underlying processes of COVID-19, since cardiovascular involvement is now recognized as a risk factor for death.

In cases of cardiovascular disorders, regardless of the presence of COVID-19, endothelial cells—like other structural cells—can produce microvesicles rich in circulating phospholipids when they are physiologically active or injured. Recipient cells may be affected either locally or systemically by these microvesicles [8]. Proteins, lipids, and nucleic acids (DNA, mRNA, microRNA, and lncRNA) are some of the components of the parent cells that are found in vesicles called exosomes. Circulating mRNA may stand in for cells that are not actually in circulation, since exosomes might disperse due to necrotic or apoptotic processes caused by damage to the vascular endothelium [9]. The term "liquid biopsy" describes the procedure of collecting cells in circulation, nucleic acids, and extracellular vesicles for molecular testing.

## 2. Mechanisms of Viral-Induced Cardiovascular Damage

**Figure 1:**
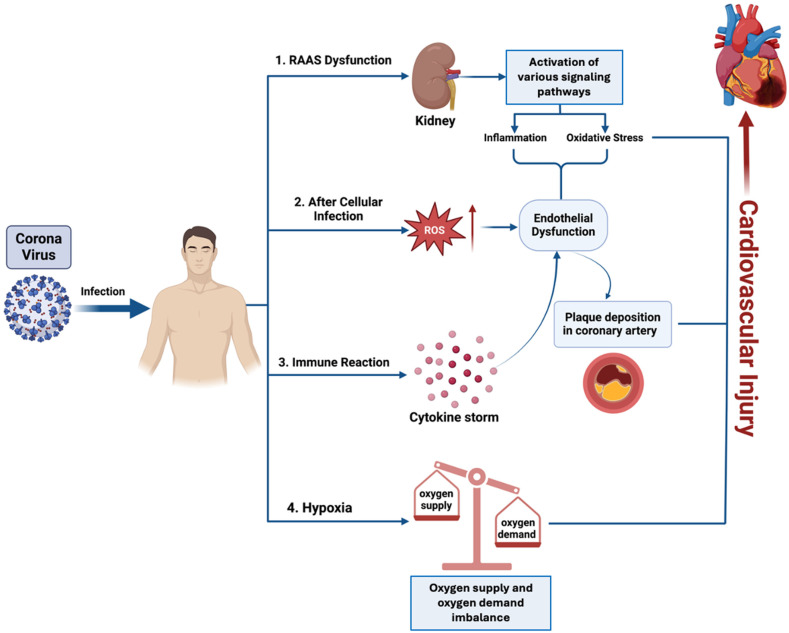
The multi-step process of coronavirus infection that can lead to cardiovascular injury. Following infection, the virus induces RAAS (renin–angiotensin–aldosterone system) dysfunction, leading to inflammation and oxidative stress, especially in the kidneys. These events contribute to endothelial dysfunction, with the generation of reactive oxygen species (ROS) resulting in plaque deposition in coronary arteries. Concurrently, the immune system’s overreaction causes a cytokine storm, which further exacerbates cardiovascular stress. The combined effects of hypoxia and the imbalance between oxygen supply and demand aggravate the risk of cardiovascular injury.

The relationship between immune responses, particularly the cytokine storm, and clinical outcomes like heart failure, arrhythmias, and myocardial injury in COVID-19 patients involves intricate mechanisms that significantly amplify cardiovascular damage. The cytokine storm, a hallmark of severe COVID-19, is characterized by the uncontrolled release of pro-inflammatory cytokines such as tumor necrosis factor-alpha (TNF-α), interleukin-1 beta (IL-1β), and interleukin-6 (IL-6) [10]. This exaggerated immune response leads to widespread inflammation, endothelial dysfunction, oxidative stress, and myocardial injury, all of which collectively contribute to severe cardiovascular complications [11] (Figure 1).

### 2.1. Cytokine Storm and Endothelial Dysfunction

The endothelium, which lines blood vessels, plays a critical role in maintaining vascular homeostasis by regulating vasodilation, thrombosis, and inflammation [12]. During a cytokine storm, inflammatory mediators disrupt the endothelial integrity, leading to endothelial dysfunction and endothelialitis [13]. Cytokines like TNF-α and IL-1β weaken inter-endothelial junctions, increasing vascular permeability. This leakage allows inflammatory cells and cytokines to infiltrate surrounding tissues, exacerbating local inflammation and further damaging vascular structures [14]. 

Endothelial dysfunction is compounded by a shift toward a pro-thrombotic state, where pro-inflammatory cytokines activate the coagulation cascade and reduce anticoagulant mechanisms, such as downregulation of thrombomodulin and tissue factor pathway inhibitors [15]. These changes, in combination with platelet activation and the formation of neutrophil extracellular traps (NETs), promote microvascular thrombosis, which can lead to ischemic injuries in various organs, including the heart. Elevated levels of D-dimer and fibrin degradation products in COVID-19 patients indicate this hypercoagulable state, contributing to myocardial infarction and other thrombotic complications [16].

### 2.2. Oxidative Stress and Cardiovascular Damage

Reactive oxygen species (ROS) production is a critical mediator of cardiovascular damage during the cytokine storm. Cytokines such as IL-6 and TNF-α stimulate immune cells, including macrophages and neutrophils, to release ROS. Excessive ROS generation overwhelms antioxidant defenses, leading to oxidative stress [17]. This environment reduces nitric oxide (NO) bioavailability, impairing vasodilation and promoting vasoconstriction, which results in increased vascular resistance and contributes to hypertension and reduced coronary perfusion [4].

ROS also directly damage endothelial cells and cardiomyocytes by inducing lipid peroxidation, DNA damage, and protein modifications [18]. This cellular injury triggers apoptosis and necrosis, further exacerbating tissue damage and impairing cardiac function. Oxidative stress amplifies the inflammatory response by activating redox-sensitive transcription factors like NF-κB, creating a vicious cycle of inflammation and tissue injury [19].

### 2.3. Cytokine Effects on Myocardial Tissue

In the myocardium, cytokines exert both direct and indirect effects that lead to structural and functional damage. TNF-α and IL-1β promote cardiomyocyte apoptosis through receptor-mediated pathways involving caspase activation [20]. Additionally, these cytokines upregulate inducible nitric oxide synthase (iNOS), increasing NO production. However, at high concentrations, NO becomes cytotoxic, contributing to further damage. IL-6, a key mediator in COVID-19, is associated with myocardial hypertrophy and adverse remodeling due to its pro-fibrotic effects on cardiac fibroblasts [21].

Indirectly, cytokines impair myocardial oxygen delivery by promoting microvascular thrombosis, which reduces coronary blood flow [22]. Additionally, systemic hypoxia caused by acute respiratory distress syndrome (ARDS) in severe COVID-19 further compromises myocardial oxygenation, leading to ischemia and injury. Elevated levels of cardiac biomarkers such as troponin (cTn), NT-proBNP, and CK-MB in COVID-19 patients reflect this myocardial damage and are strong predictors of mortality [23].

### 2.4. Mechanisms Leading to Specific Clinical Outcomes

#### 2.4.1. Heart Failure

COVID-19 is closely linked with heart failure (HF), both as a pre-existing condition that worsens outcomes and as a complication of the disease itself [24]. Approximately one-third of hospitalized COVID-19 patients with prior HF experience acute decompensation, while others develop new-onset HF [25]. For example, in an Italian study, 9.1% of hospitalized COVID-19 patients developed acute HF, nearly half of whom had no prior HF history [26]. Acute HF was associated with a significantly higher mortality rate, as seen in a Spanish cohort where patients with HF had a 46.8% mortality rate compared with 19.7% in those without HF [27].

Long-term consequences of COVID-19 on the heart, such as persistent myocardial damage and fibrotic changes, suggest that HF may develop as a chronic condition in survivors [28]. Imaging studies, including echocardiography and cardiac MRI, have identified structural and functional cardiac changes up to months after recovery, underscoring the need for ongoing cardiovascular monitoring and management in COVID-19 patients [29].

#### 2.4.2. Arrhythmias

COVID-19 is associated with various arrhythmias and disruptions to the cardiac electrical conduction system [30]. Sinus tachycardia is the most common tachycardia in hospitalized patients, followed by atrial fibrillation (AF), which occurs in about 10–18% of cases. Studies suggest that AF may increase in-hospital mortality, with one large analysis reporting a 5.4% new-onset AF rate and significantly higher mortality (45.2% vs. 11.9%) [31]. However, after adjusting for factors like demographics, comorbidities, and disease severity, this association was not statistically significant. Other studies found similar trends, with new-onset AF linked to higher intra-hospital mortality rates [32].

Ventricular arrhythmias are also reported, including non-sustained ventricular tachycardia (15.6%) and rare cases of sustained ventricular tachycardia and fibrillation, particularly in patients with elevated troponin levels [33]. Additional complications include cardiac arrests, bradyarrhythmias, and conduction abnormalities such as changes in ventricular repolarization markers on ECG. Arrhythmias in COVID-19 patients are likely triggered by myocardial injury (e.g., myocarditis or acute coronary syndrome), systemic hypoxemia, inflammation, or severe acute illness, underscoring the complex cardiovascular impact of the disease [34].

#### 2.4.3. Myocardial Infarction

COVID-19-related myocarditis results from a combination of direct viral effects, endothelial dysfunction, and systemic inflammation, which together contribute to myocardial injury and infarction [35]. SARS-CoV-2 directly infects cardiomyocytes and endothelial cells by binding to the ACE2 receptor, which is highly expressed in cardiac tissue and blood vessels [36]. Following receptor binding, the viral spike protein is primed by TMPRSS2, allowing viral entry and replication. This process can lead to direct cytopathic effects, including myocardial necrosis and dysfunction, though the extent of ACE2 expression in cardiomyocytes remains debated [4]. Additionally, SARS-CoV-2 infects endothelial cells of coronary vessels, causing localized inflammation and endothelial dysfunction [37]. Viral invasion triggers macrophage recruitment, complement activation, and apoptosis in these areas, further damaging the vascular endothelium and potentially leading to thrombus formation [30].

Systemic inflammation also plays a central role, with elevated cytokines such as interleukin-6 (IL-6) and tumor necrosis factor-alpha (TNF-α) driving a “cytokine storm”. This hyperinflammatory response leads to extensive tissue damage, including myocarditis [38]. IL-6 is particularly significant, as it recruits inflammatory cells to the myocardium and exacerbates myocardial necrosis. The prolonged inflammatory state also increases the risk of thrombus formation within coronary vessels due to platelet activation and elevated clotting factors [39]. In severe cases, the cytokine storm may aggravate pre-existing myocarditis, compounding the myocardial damage. Together, these interconnected mechanisms highlight the multifactorial pathways involved in COVID-19-related myocarditis and myocardial infarction, involving viral entry, immune-mediated inflammation, and vascular injury [40].

#### 2.4.4. Thromboembolism in COVID-19 Patients

Several mechanisms underlying the increased thromboembolic risk in COVID-19 have been identified. SARS-CoV-2 enters host cells through ACE2, leading to its internalization and degradation, which subsequently increases serum angiotensin II levels [41]. This elevation not only promotes vasoconstriction but also triggers pro-inflammatory and pro-thrombotic effects. Dysregulation of the renin–angiotensin–aldosterone system (RAAS) further exacerbates endothelial dysfunction, creating conditions conducive to coagulopathy in COVID-19 patients [42]. Supporting this, studies have revealed perivascular T-cell infiltration, endothelial injury, and widespread pulmonary vessel thrombosis in patients who succumbed to COVID-19 [43,44]. Notably, alveolar capillary microthrombi were found to be nine times more prevalent in COVID-19 patients compared with those with severe influenza, underscoring the virus’s significant pro-thrombotic potential.

COVID-19-associated endotheliopathy has also been linked to increases in von Willebrand factor (vWF) release, platelet activation, and hypercoagulability, which collectively drive venous, arterial, and microvascular thrombosis [45]. In critically ill COVID-19 patients, elevated levels and activity of vWF, soluble P-selectin (a marker of endothelial and platelet activation), soluble CD40L, and factor VIII have been observed. These markers, particularly vWF and thrombomodulin, correlate with mortality in ICU settings. Interestingly, even non-critically-ill COVID-19 patients exhibit increased vWF levels compared with healthy controls, highlighting the systemic nature of COVID-19-induced coagulopathy [45].

### 2.5. Evidence Supporting the Link Between Immune Responses and Cardiovascular Outcomes

Histopathological studies of post-mortem COVID-19 cases have revealed extensive endothelial damage, microvascular thrombosis, and inflammatory cell infiltration in the myocardium [4]. Intussusceptive angiogenesis, a hallmark of severe endothelial injury, has been observed in these cases, reflecting the profound vascular remodeling induced by the cytokine storm. These findings correlate with clinical observations of elevated cardiac biomarkers and echocardiographic evidence of reduced left ventricular ejection fraction in severe cases [46].

In conclusion, the cytokine storm and resulting immune dysregulation in COVID-19 patients lead to a cascade of events that culminate in cardiovascular complications [47,48]. These mechanisms involve endothelial dysfunction, oxidative stress, direct myocardial injury, and vascular remodeling. Understanding these pathways provides a foundation for targeted therapies such as cytokine inhibitors and anticoagulants to mitigate cardiovascular damage and improve clinical outcomes in affected patients [48].

### 2.6. Chronic Viral Infections and Long-Term Cardiovascular Risks

There is a reciprocal relationship between viral infections and cardiovascular disease (CVD): CVD risk increases with viral infection, and those who already have CVD are more vulnerable to severe viral infection. Acute respiratory and urinary tract infections, bacteremia, and sepsis have been linked to an elevated risk of myocardial infarction and stroke [49,50]. Evidence from randomized controlled trials shows that influenza vaccination lowers the risk of cardiovascular events, which corroborates these conclusions [51]. Even while the risk of cardiovascular disease seems to peak just after infection, it can continue to rise for years [52]. However, the complete spectrum of infectious disease events’ contribution to the incidence of cardiovascular disease events has not been assessed by much large-scale research. 

Since infectious diseases are widespread, they may have a significant impact on the burden of cardiovascular disease [53]; however, it is unknown what percentage of cardiovascular events may be related to all infections. The acute phase of an infection can raise the short-term risk of cardiovascular events through several methods. Examples of factors that may contribute to electrical instability of the heart, atherosclerotic plaque instability, and a concomitant hypercoagulable state include the activation of inflammatory molecules and platelets, increased inflammation in atheromatous plaques, endothelial dysfunction, and enhanced sympathetic nervous system activity with catecholamine release [54]. The cardiovascular system is likely to be more affected by infections in the short term than in the long term, despite the fact that severe infections have been associated with an elevated risk of cardiovascular disease after the acute phase [55]. According to recent studies, chronic viral infections—like those brought on by the cytomegalovirus (CMV), hepatitis C virus (HCV), and human immunodeficiency virus (HIV)—may make these cardiovascular risks worse by impairing endothelial function and promoting immune dysregulation and chronic inflammation [56].

## 3. Role of Genetic Factors in Viral-Related CVD

A type 1 transmembrane protein called angiotensin-converting enzyme 2 (ACE2) is present on the surface of human cells, and SARS-CoV-2 binds to it to enter human cells. Spike protein proteolysis is initiated upon connection to ACE2 by cellular proteases, such as the transmembrane protease serine 2. The virus triggers an endosome-dependent process in order to infect host cells and multiply itself [57]. Inside the host cell, SARS-CoV-2 utilizes the cell’s machinery to synthesize viral proteins and replicate its genetic material.

ACE2 has the capacity to enzymatically convert angiotensin II into angiotensin-(1–7) and metabolize angiotensin I (Ang I), resulting in the production of angiotensin-(1–9), which promotes vasodilation and exerts anti-inflammatory, antioxidant, and cardioprotective effects. Their expression is downregulated by SARS-CoV-2. Hence, there is an increase in the level of angiotensin II, leading to endothelial dysfunction, chronic myocardial hypoxia, and cardiomyocyte death [57].

Alveolar tissue, cardiomyocytes, pericytes, fibroblasts, endothelial cells, and smooth muscle cells all have significant levels of ACE2 expression [58].

There is some evidence from in vitro investigations that SARS-CoV-2 can infect the heart. The virus may infect cardiomyocytes produced from human induced pluripotent stem cells through ACE2- and cathepsin-mediated processes, according to Sharma et al., who also showed that within 72 h, the virus can trigger apoptosis [59]. Many studies emphasize a mechanism involving direct myocardial injury mediated by ACE2, which could potentially serve as a therapeutic target. Small in vitro studies also proposed the hypothesis that ACEIs play a significant role in counterbalancing proinflammatory responses, promoting an upregulation in the expression of the interleukin-1 receptor type 2 and RETN gene (inhibitors of proinflammatory cytokines) in the monocytes of these patients [60].

A study investigated how ACE2 genetic variations influence SARS-CoV-2 susceptibility and their link to cardiovascular diseases. ACE2, the receptor for SARS-CoV-2, facilitates viral entry and regulates cardiovascular health. The D355N variant significantly reduces binding to the virus’s spike protein, lowering infection risk in lab and animal models. ACE2 variants are also associated with conditions like hypertension and heart disease, which can worsen COVID-19 outcomes [61].

Differences in ACE2 variants across populations may explain variations in COVID-19 severity. The D355N mutation disrupts critical structural features of ACE2, reducing infection risk while possibly affecting cardiovascular functions. These findings emphasize the role of ACE2 variants in COVID-19 outcomes and the need for personalized medical strategies [62,63].

Another study explored the association between genetic variants of the ACE2 gene, specifically the rs2074192 polymorphism, and their implications for cardiovascular diseases and COVID-19 severity in an Indonesian population. It highlighted that variations in the ACE2 gene can influence blood pressure regulation and may contribute to the pathogenesis of cardiovascular conditions, which in turn can exacerbate the severity of COVID-19. The findings suggest that individuals with certain ACE2 genotypes, particularly those with hypertension, are at a higher risk for severe COVID-19 outcomes, emphasizing the importance of understanding genetic factors in managing both cardiovascular diseases and viral infections [64].

Genetic variations in ACE2 also affect its expression, influencing viral entry and cardiovascular risks like hypertension and heart failure. For instance, a study examined genetic variations in the ACE2 gene and their impact on COVID-19 susceptibility. ACE2 plays a key role in viral entry and is linked to cardiovascular conditions. The research identified 299 ACE2 variants, with 5 potentially reducing viral binding, possibly resisting infection. While the findings are promising, the lack of case-control data calls for further investigation. Future research should explore ACE2’s functional effects in diverse populations and its relationship with both COVID-19 and cardiovascular disease [65].

The most often observed laboratory abnormalities associated with COVID-19 coagulopathy are a decreased platelet count, a slightly prolonged clotting time, higher concentrations of D-dimer and fibrinogen, and a lower platelet count [66]. It is common for fibrinogen level to be high during the start of a COVID-19 infection, but it starts to drop in the latter stages of the disease in those who do not survive [67]. It has been consistently reported that D-dimer levels significantly increase in COVID-19 individuals, whereas activated partial thromboplastin time (APTT) and prothrombin time (PTT) show a little extension in these patients [68]. There is mounting evidence that people infected with COVID-19 often have high levels of the coagulation marker D-dimer [69,70].

Moreover, a study highlighted the genetic mechanisms linking SARS-CoV-2 infection to cardiovascular injuries. Three core genes—SON, OGT, and RORA—were identified as shared across cardiovascular conditions and SARS-CoV-2-infected lung cells, suggesting their role in bridging lung damage and systemic cardiovascular injuries. SON regulates the cell cycle and RNA splicing, OGT modulates stress responses with cardioprotective effects, and RORA reduces inflammation and prevents myocardial damage [71].

Additionally, RPS29 and SPAG9 were implicated in immune responses and potential cardiac damage, with RPS29 enhancing immune cell recruitment and SPAG9 potentially inducing autoantibodies against cardiac proteins [72,73]. Four cardioprotective genes—NDUFA4L2, NDUFB7, MRPS11, and HIKESHI—were downregulated in infected cardiomyocytes, contributing to mitochondrial dysfunction and increased cardiac stress [71].

These findings reveal potential genetic targets for understanding and treating COVID-19-related cardiovascular complications, though experimental validation is needed to confirm these results.

## 4. Epigenetic Modifications and Cardiovascular Vulnerability

Viruses use epigenetic mechanisms, specifically CpG methylation, to undergo syncytium formation and endocytosis. The virus either stimulates host cell–cell fusion or fuses with the host’s cell membrane as it spreads. Together with the invasion of nearby cells and innate antiviral immune system evasion, both activities encourage virus endocytosis [74]. Syncytiums, which are created during membrane–virus or cell–cell fusion, are typically linked to CoVs [75]. Syncytin genes pose a risk for several disorders, since they are hypomethylated and highly active in the mammalian placenta and hypermethylated and muted in other tissues [76]. According to epigenetic studies, DNA and RNA viruses create an environment that is conducive to their replication and spread by altering host metabolism and gene expression, which results in the development of antagonistic regulatory roles [77]. CoVs promote epigenetic changes by inhibiting the host’s antigen presentation or the activation of interferon-response genes [78]. Additionally, it has been shown that the rate of ACE2 gene synthesis is epigenetically controlled [79]. 

ACE2 has been linked to the development of cardiovascular illnesses as well as the regulation of cardiac structure and function [80,81,82]. Myocardial fibrosis and left ventricular hypertrophy are prevented by overexpressing ACE2, which is advantageous for cardiac function [83,84]. One way that ACE2 helps the heart is by hydrolyzing Ang II into Ang 1-7 [85]. This is because Ang II causes cardiovascular illnesses, whose progression is stopped by blocking Ang II. In contrast, ACE2 deficiency and increased intracardiac Ang II levels, on the other hand, produce cardiac remodeling and dysfunction due to pressure overload [86]. 

Angiotensin-converting enzyme 2 (ACE2) is a receptor that the virus SARS-CoV-2 uses to enter host cells. ACE2 is essential for the renin–angiotensin–aldosterone system (RAAS) because it degrades angiotensin II (ANG II), a peptide that can cause cardiovascular problems. Viral infection-induced ACE2 internalization can result in lower ACE2 levels and higher ANG II, which can worsen cardiovascular diseases. Moreover, individuals who already have cardiovascular disease are more vulnerable to serious consequences from COVID-19. Viral infection-induced ACE2 function reduction may deteriorate heart health and raise the risk of acute cardiovascular events, which may be concerning for patients with conditions such as hypertension and heart failure [87].

Increasing histone acetylation in genes regulating pro-inflammatory cytokines like TNF-α and IL-6 was reported in a group of severe COVID-19 patients. This observation was linked with increased endothelial dysfunction and systemic inflammation. According to these results, virally induced changes to histones may worsen cardiovascular consequences by encouraging a pro-inflammatory environment [88].

Although comprehensive research on the cardiac epigenome in COVID-19 patients is lacking, the limited research available shows altered epigenetic markers, such as DNA methylation, causing changes in the expression of several genes. Histone H3 citrullination, an alteration that helps identify neutrophil extracellular traps (NETs), is elevated in COVID-19 patients [89]. The body’s first line of defense against SARS-CoV-2 infection is the neuroendocrine tumor suppressors (NETs), which is why heme changes, and the immune response are so closely connected. Some researchers found that high levels of circulating histone–DNA complexes and histone H3 cleavage were linked to severe cases of COVID-19 [90,91]. According to certain studies, histone changes such as H3K4me3 enhance transcription in response to SARS-CoV-2 infection. This is especially true with interferon response genes. Researchers have also connected the SARS-CoV-2 infection cycle to components of the SWI/SNF chromatin remodeling complex [92].

A major actor in epigenetic alterations is non-coding RNA (nc-RNA), which also plays a role in DNA methylation. According to research by some scientists, COVID-19 can up- or downregulate certain nc-RNAs, which might indicate their potential as disease biomarkers. Notable non-coding RNAs (nc-RNAs) investigated here include miR-30b, linked to ischemic heart disease, and miR-29s, which exhibits anti-fibrotic properties in the heart [93]. Severe adverse cardiac consequences, including heart failure, myocarditis, pericarditis, and myocardial infarction, have been demonstrated by several investigations into the epigenetics of COVID-19 [94,95].

## 5. Genetic and Epigenetic Modulation of Cardiovascular Outcomes

**Figure 2:**
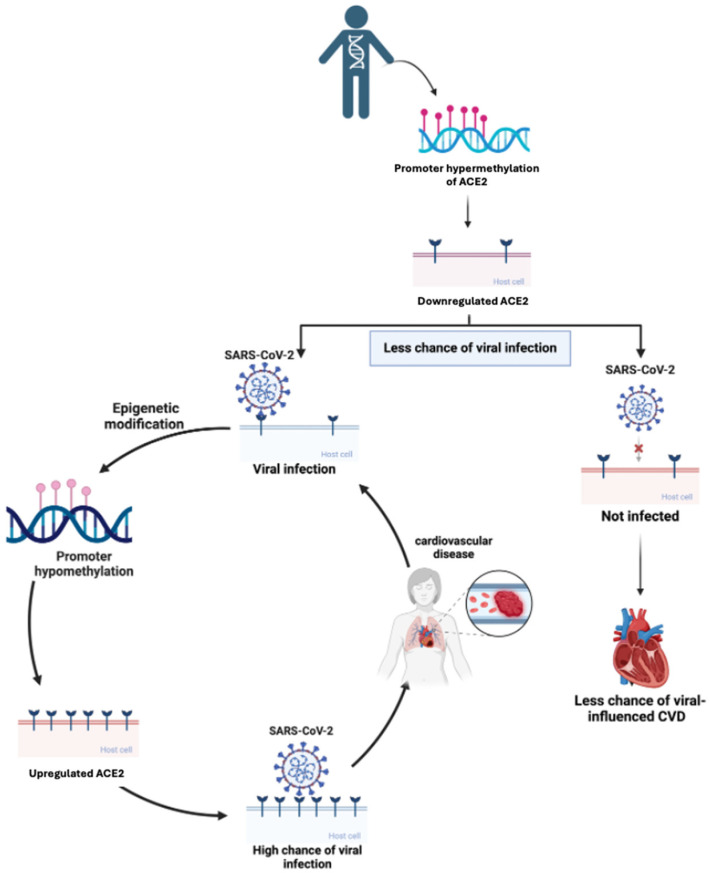
How epigenetic modifications affect ACE2 expression and influence viral infection and cardiovascular disease (CVD) risk. Promoter hypermethylation downregulates ACE2, reducing the chance of viral infection, while promoter hypomethylation leads to ACE2 upregulation, increasing susceptibility to viral entry. This elevated risk can result in viral-induced cardiovascular diseases, completing a cycle where epigenetic factors shape both viral infection susceptibility and CVD progression.

Genetic and epigenetic variables that affect a person’s vulnerability to serious illness and cardiovascular problems can be partly responsible for the variation in cardiovascular outcomes among COVID-19 patients. Variations in ACE2 expression, a gene implicated in the renin–angiotensin–aldosterone system (RAAS), may influence a person’s susceptibility to cardiovascular problems brought on by COVID-19 [96]. For instance, specific ACE2 gene variations have been linked to varying susceptibilities to SARS-CoV-2 infection and the severity of cardiovascular consequences such as myocarditis and thrombosis. Furthermore, prothrombin or factor V Leiden gene variants3w, which are genetically predisposed to hypercoagulability, may raise the risk of thromboembolic events in COVID-19 patients [97]. Interaction between genetic and epigenetic factors in CVD outcomes is depicted in Figure 2. 

In COVID-19, epigenetic modifications such as DNA methylation, histone modification, and microRNA expression can potentially influence cardiovascular outcomes. For example, it has been demonstrated that SARS-CoV-2 infection alters the expression of microRNAs that control the coagulation and inflammatory pathways. These changes in epigenetics can modify the host’s immunological response, which may exacerbate cardiovascular problems [98]. Furthermore, in the context of COVID-19, those who already have epigenetic changes associated with cardiovascular risk, such as those brought on by environmental factors like smoking or obesity, may be more vulnerable to serious cardiovascular consequences [96].

A study explored the genetic and epigenetic interplay in viral-related cardiovascular diseases (CVDs), particularly SARS-CoV-2, highlighting its manipulation of host signaling pathways such as NF-κB, IL-17, and HIF-1. Genetic predispositions like immune pathway polymorphisms amplify susceptibility to severe CVD outcomes. Epigenetic modifications, including upregulated miRNAs (e.g., hsa-miR-429, hsa-miR-1286) and altered transcription factors (e.g., SMAD3, STAT1), disrupt inflammation and endothelial function, worsening disease severity [99].

Key pathways such as HIF-1 signaling are hijacked to promote endothelial proliferation and thrombosis, while aberrant IL-17 signaling exacerbates inflammation, contributing to complications like pulmonary hypertension and acute lung injury. The study underscores how these molecular interactions shape CVD progression in viral infections and identifies potential therapeutic targets in miRNA regulation and epigenetic modulation to mitigate risks [99].

### Genetic and Epigenetic Interaction in CVD 

An individual’s cardiovascular outcome after being exposed to a virus is significantly influenced by the interplay between genetic predispositions and epigenetic alterations. For example, those who have genetic abnormalities that make them more prone to thrombosis may also have epigenetic modifications that worsen inflammation or affect endothelial function, which could lead to more severe thromboembolic events [100].

Genetic variations and epigenetic variables like DNA methylation both affect the expression of ACE2, the entrance receptor for SARS-CoV-2. Epigenetic alterations that downregulate ACE2 expression may provide protection against severe COVID-19-related cardiovascular problems in persons with specific ACE2 polymorphisms. This is because they limit the entry of the virus into heart and lung tissues. On the other hand, hypomethylation of ACE2 may increase the severity of viral infection and the related cardiovascular implications, such as myocarditis and heart failure, among people with genetic predispositions to higher ACE2 expression [101].

SARS-CoV-2 infection disrupts key molecular pathways, leading to cardiovascular damage through genetic predispositions and epigenetic modifications. A central mechanism involves the dysregulation of the ACE2/Ang-(1-7)/Mas axis, a protective component of the renin–angiotensin system (RAS). The virus downregulates ACE2, tipping the balance toward the deleterious ACE/Ang II/AT1 axis, which promotes vasoconstriction, inflammation, fibrosis, and oxidative stress, ultimately causing cardiac remodeling and failure. Epigenetic changes also play a significant role, with altered DNA methylation observed in key genes such as Peg10, which is hypomethylated and upregulated, driving cell proliferation, and Ece1, which is hypermethylated, leading to its dysregulation and associated heart defect [102]. 

Long-term cardiovascular outcomes in the context of long COVID may be influenced by the interaction between virally induced epigenetic modifications and genetic predispositions to CVD, such as polymorphisms in coagulation or inflammatory genes. For instance, months after the initial infection, patients with a genetic predisposition to hypercoagulability who also show epigenetic changes that heighten inflammation may be more vulnerable to long-lasting cardiovascular problems like arrhythmias or heart failure [103].

Severe acute respiratory syndrome coronavirus 2 (SARS-CoV-2) infection has been linked to multisystemic involvement, encompassing the cardiovascular and cerebrovascular systems in addition to pulmonary involvement [104]. The myocardial, pericardium, and conduction systems may sustain damage due to direct viral invasion, inflammation, and immune responses [105]. Coronavirus disease 2019 (COVID-19) patients have similar neurologic symptoms that have been attributed to postinfectious immunological reactions, hypercoagulability, endotheliopathy, and direct neuroinvasion. Long-term consequences may arise if this damage persists [106]. Even months after the initial infection has cleared up, long COVID has been linked to several enduring symptoms, including cardiovascular problems. These chronic cardiovascular side effects include heart failure, arrhythmias, myocarditis, and malfunction of the autonomic nervous system [107].

After recovering from acute COVID-19, some patients have long-term heart problems. Studies using magnetic resonance imaging (MRI) have shown that some patients still exhibit myocardial fibrosis and inflammation months after they have recovered, which suggests that prolonged COVID-19 may put people at risk for long-term cardiac problems, such as heart failure [107,108].

Persistent arrhythmias, such as ventricular tachycardia and atrial fibrillation, are common in long-term COVID-19 patients. These arrhythmias raise the risk of sudden cardiac death, palpitations, and dizziness. Furthermore, patients with prolonged COVID-19 frequently experience autonomic dysfunction, which is defined by dysregulated blood pressure and heart rate regulation. This condition may be related to chronic inflammation and damage to the autonomic nervous system [109].

## 6. Role of Viral Persistence and Immune Activation in CVD Development

**Figure 3:**
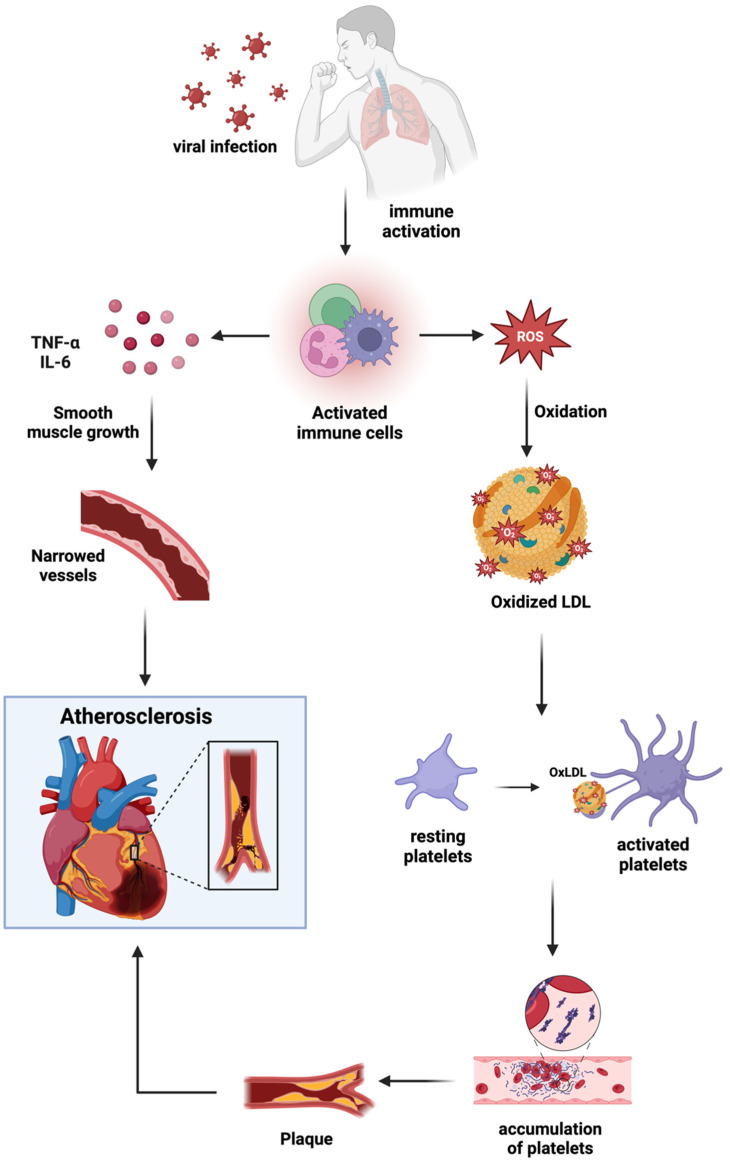
The progression from viral infection to the development of atherosclerosis through immune system activation. The viral infection triggers immune cells to release cytokines like TNF-α and IL-6, promoting smooth muscle growth and vessel narrowing. Activated immune cells generate reactive oxygen species (ROS), which oxidize low-density lipoproteins (LDLs), leading to the formation of oxidized LDLs (OxLDLs). These OxLDLs activate platelets, resulting in platelet aggregation and the formation of plaque. The accumulation of these plaques contributes to the development of atherosclerosis, increasing the risk of cardiovascular disease.

Even in patients receiving antiviral therapy, there are still viral reservoirs in the body, which leads to continued immunological activation and is a primary cause of CVD in these people [110]. Reactive oxygen species (ROS) are produced in excess because of persistent immunological activation, which damages the endothelium and encourages atherosclerosis [111,112]. Moreover, ROS oxidize low-density lipoprotein (LDL), which promotes macrophage uptake of oxidized LDL and contributes to the development of plaque [113]. Viral reservoirs contribute to a loop of inflammation and immunological activation that sustains cardiovascular risk, especially in people with COVID-19 [114]. The steps of progression from viral infection to the development of atherosclerosis via immune system activation is represented in Figure 3.

By encouraging inflammation and macrophage infiltration into vascular tissues, immune dysregulation speeds up the development of atherosclerosis. Pro-inflammatory cytokines like TNF-α and IL-6 promote smooth muscle cell growth in the artery walls, thickening the vessel walls and narrowing the arteries in the process [110].

## 7. Antiviral Therapies

Despite the significant improvements in viral suppression and patient outcomes brought about by antiviral medicines, including antiretroviral therapies (ARTs) and direct-acting antivirals (DAAs), immunological dysregulation and the related cardiovascular risks remain a concern [115]. For instance, long-term use of several antiviral medications has been connected to metabolic problems such as insulin resistance and dyslipidemia in viral infected patients receiving ARTs, both of which raise the risk of CVD [116]. Similarly, due to the long-term consequences of immunological dysregulation and endothelial dysfunction, individuals who have been cured of COVID-19 with DAAs may, however, show an increased risk of CVD [115,117].

COVID-19 treatments, including antiviral and anti-inflammatory drugs, carry significant risks of cardiovascular complications, driven by a range of direct and indirect mechanisms. Remdesivir, a viral RNA polymerase inhibitor, is linked to bradycardia, hypotension, and QT prolongation [118]. These effects may stem from its interference with potassium ion channels and modulation of autonomic pathways, which can disrupt normal electrical conduction in the heart and lead to arrhythmias or myocardial stress. Lopinavir/ritonavir, originally developed for viral infection treatment, contributes to QT and PR interval prolongation, arrhythmias, and torsades de pointes through its inhibition of cytochrome P450 3A4 [28]. This inhibition leads to altered drug metabolism, accumulation of cardiotoxic substances, and impaired cardiomyocyte function, compounded by its disruption of the PI3K/Akt signaling pathways, which are crucial for cellular survival and energy metabolism [119].

Hydroxychloroquine (HCQ) and chloroquine (CQ), used for their antiviral and anti-inflammatory properties, inhibit potassium and sodium currents, causing QT prolongation, QRS widening, and increased arrhythmogenic potential [120,121]. HCQ also induces inflammatory cytokine-mediated channelopathies, further heightening the risk of arrhythmias [122]. These effects are exacerbated when combined with azithromycin, which independently prolongs the QT interval. Ribavirin, a broad-spectrum antiviral, interferes with mitochondrial function, promoting calcium metabolism disorders and energy deficits in cardiomyocytes, which can result in bradycardia, dyspnea, and chest pain. Long-term or high-dose use is particularly dangerous for patients with pre-existing cardiovascular conditions [123].

Anti-inflammatory therapies, such as tocilizumab, an interleukin-6 (IL-6) receptor inhibitor, and dexamethasone, a corticosteroid, are used to control cytokine storms but can have cardiotoxic effects [124]. Tocilizumab suppresses IL-6 activity but may induce mitochondrial oxidative stress, cardiomyocyte apoptosis, and endothelial dysfunction, manifesting as hypertension, arrhythmias, and myocardial injury [125]. Dexamethasone, while reducing inflammation, can cause fluid retention, electrolyte imbalances, and arrhythmias, especially at higher doses [126]. Interferon-alpha (IFN-α), used for its antiviral properties, is associated with ischemic cardiomyopathy and endothelial damage due to its pro-inflammatory effects and promotion of oxidative stress in vascular tissues [123].

Drug–drug interactions further amplify risks, particularly with Paxlovid (nirmatrelvir/ritonavir), which inhibits cytochrome P450 enzymes and can cause bradycardia or hypertension by increasing plasma levels of other co-administered drugs [127]. Combinations of three or more antiviral agents heighten the likelihood of severe adverse effects, including cardiotoxicity and metabolic disturbances [128].

These findings highlight the complex interplay between COVID-19, its treatments, and cardiovascular health. Personalized risk assessments, vigilant cardiac monitoring, and cautious use of combination therapies are critical to minimizing adverse outcomes. Advanced drug development strategies, such as AI-driven approaches, may help reduce these risks by designing therapies with fewer off-target effects on the cardiovascular system.

## 8. Screening for Cardiovascular Risk Prediction

**Figure 4:**
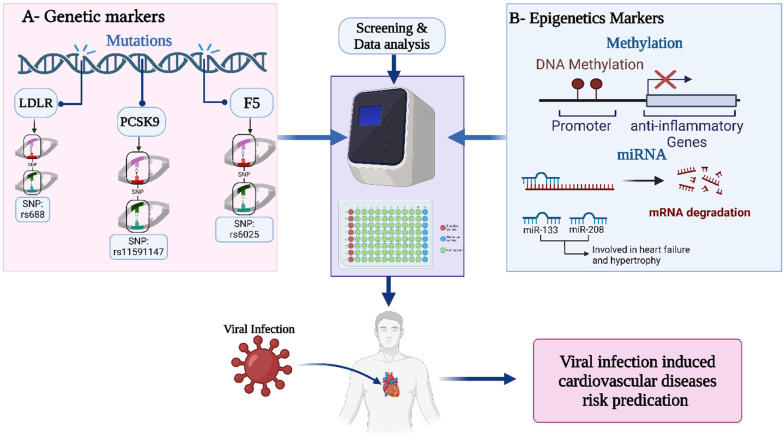
The relationship between genetic and epigenetic markers in predicting cardiovascular disease (CVD) risk due to viral infections. Part A focuses on genetic markers like LDLR, PCSK9, and F5, highlighting specific mutations (SNPs: rs688, rs11591147, rs6025) that contribute to CVD risk. Part B showcases epigenetic factors such as DNA methylation and miRNA regulation (e.g., miR-133, miR-208), which influence gene expression related to inflammation and heart failure. The integration of genetic and epigenetic data facilitates screening and analysis to predict viral infection-induced CVD risk.

A person’s genetic predisposition is a major factor in predicting their risk of cardiovascular disease, particularly when viral infections are present. A patient’s innate predisposition to heart disease can be discerned through genetic screening for markers such as single nucleotide polymorphisms (SNPs) and mutations in genes such as the low-density lipoprotein receptor gene (LDLR), factor V Leiden mutation (F5), and others. These genes control vital functions that are directly impacted by viral infections, including inflammation, thrombosis, and cholesterol metabolism [112]. For example, individuals with genetic variations linked to hypercholesterolemia have an increased risk of developing atherosclerosis; additionally, a viral infection may aggravate the underlying illness and cause acute cardiovascular events. Furthermore, the degree of the inflammatory response during a viral infection can be influenced by genetic polymorphisms in immune response genes, raising the risk of myocarditis or other heart-related problems [129,130]. Different genetic and epigenetic screening markers are shown in Figure 4.

In addition to genetic changes, epigenetic modifications, such as DNA methylation, histone acetylation, and control over non-coding RNAs like microRNAs, have a substantial impact on how genes are expressed in response to cardiovascular stress and viral infections. Epigenetic alterations brought on by viral infections have been shown to worsen or even induce cardiovascular illnesses [131].

Screening for cardiovascular risk in individuals with COVID-19 is crucial due to the virus’s potential to exacerbate existing heart conditions and induce new cardiovascular complications [29]. Key risk factors include pre-existing cardiovascular diseases, hypertension, diabetes, obesity, chronic kidney disease, smoking, a sedentary lifestyle, older age, male sex, and a family history of premature cardiovascular disease [132]. Elevated levels of biomarkers such as troponin, D-dimer, C-reactive protein (CRP), B-type natriuretic peptide (BNP), or N-terminal pro-BNP (NT-proBNP) and ferritin have been associated with worse outcomes in COVID-19 patients [133]. 

Recent studies have highlighted the prognostic value of these biomarkers. For instance, elevated high-sensitivity cardiac troponin I (hs-cTnI) and NT-proBNP levels have been linked to increased mortality in COVID-19 patients [134]. Additionally, early detection of elevated hs-cTnI and BNP can predict mortality, underscoring the importance of systematic assessment of cardiac biomarkers in these patients [135]. 

In summary, monitoring cardiovascular risk factors and biomarkers in COVID-19 patients is essential for the early identification and management of potential complications, thereby improving patient outcomes.

## 9. Personalized Medicine Approaches Based on Genomics and Virology

Clinicians can carefully design treatment interventions for patients who are more likely to develop cardiovascular problems during viral infections owing to personalized medicine, which is based on the identification of genetic and epigenetic risk factors [136]. To avoid complications like deep vein thrombosis, pulmonary embolism, or stroke, patients with genetic markers like the F5 Leiden mutation, which predisposes them to an increased risk of thrombosis, may benefit from the early introduction of anticoagulant therapies during viral infections [137]. 

In order to avoid severe inflammatory responses that could harm the heart, those with genetic changes that raise the risk of inflammation, such as polymorphisms in the IL6 gene, may benefit from early anti-inflammatory treatment or immunomodulatory medications [138]. Furthermore, because genetic variations in drug-metabolizing enzymes such as the CYP450 enzymes might affect how well a patient metabolizes and responds to antiviral drugs, pharmacogenomics is essential in guiding antiviral therapy. Finding these differences can aid in the best possible medicine selection and dose, minimizing side effects and enhancing therapeutic results [139].

One possible method for reducing the hazards of viral infections on the heart is the use of epigenetic modulators. Epigenetic medications like DNA methylation inhibitors and histone deacetylase (HDAC) inhibitors may be able to undo the detrimental epigenetic alterations that are linked to cardiovascular disease [140]. For example, HDAC inhibitors have shown promise in lowering inflammation and fibrosis in heart tissues by restoring normal gene expression patterns that are disturbed during viral infections. These medications may be used to modify gene expression and lower the risk of serious heart damage in patients who show epigenetic modifications indicative of an elevated risk of myocarditis or heart failure [141]. Furthermore, microRNAs (miRNAs) have become significant epigenetic regulators in cardiovascular disease. One novel therapeutic technique that may be available is to target miRNAs that are dysregulated during viral infections. For example, in patients recovering from viral infections such as SARS-CoV-2, targeting specific miRNAs involved in the regulation of cardiac hypertrophy or fibrosis may prevent long-term heart damage [142].

## 10. Bioinformatic Investigation of Genetic Predisposition for CVD and COVID-19 Severity

The COVID-19 pandemic served to highlight the profound effects of the SARS-CoV-2 virus on global health systems. Cardiovascular events have emerged as a significant complication of COVID-19, prompting extensive research into the relationship between COVID-19 and CVD, particularly due to the severe cardiovascular events reported in numerous COVID-19 patients [143]. Genetic variants as contributing factors in patients’ symptoms severity can significantly influence cardiovascular events in COVID-19 patients. Numerous investigations have identified certain genetic mutations linked to increased vulnerability to severe COVID-19 and associated cardiovascular problems [107]. Studies have shown that mutations in genes related to the immunological response, including Toll-Like Receptor 7 (TLR7) and Unc-13 Homolog D (UNC13D), correlate with adverse COVID-19 consequences [107]. Moreover, genetic polymorphisms associated with cardiovascular function have been linked to the severity of COVID-19. Mutations in genes such as the heterozygous desmin gene (DES) and Striated Muscle Enriched Protein Kinase (SPEG), associated with cardiovascular disorders, have been recognized as possible risk factors for COVID-19 mortality [144]. Additionally, several SNPs linked to both COVID-19 severity and CHD have been found in several genes, including leucine zipper transcription factor-like 1 (LZTFL1), histo-blood group ABO system transferase (ABO), inflammation and lipid regulator with UBA-like and NBR1-like domains (ILRUN), and calcium channel flower domain containing 1 (CACFD1) [145]. Here are some elaborated insights into the associations between these genetic variations and CVD in individuals with COVID-19.

The TLR7 gene is a vital element of the innate immune system, essential for detecting viral RNA and triggering immunological responses [146]. Polymorphisms in TLR7 can make the patient vulnerable to viral infections such as SARS-CoV-2. The TLR7 frameshift mutation rs2042915990 (c.2129_2132del; p.[Gln710Argfs*18]) causes a loss of four nucleotides, resulting in a shift in the reading frame, generating a truncated protein that is probably non-functional [147]. This mutation is linked to severe COVID-19 instances, suggesting that persons with this variation may have compromised immune responses to SARS-CoV-2 [107]. The TLR7 missense mutation rs200553089 (c.2383G>T; p.[Val795Phe]) causes a substitution of valine with phenylalanine at position 795 (V795F) of the TLR7 protein [147]. This mutation may deleteriously influence the protein’s conformation and functionality, perhaps resulting in decreased expression and impaired signaling in response to viral infections [148]. Mutations in TLR7 result in reduced expression of the receptor in peripheral blood mononuclear cells (PBMCs), which impacts the synthesis of type I interferons (IFNs), which are essential for antiviral defense [149].

The UNC13D gene has been identified as a contributor to the onset of cytokine storms and is classified as a gene associated with hemophagocytic lymphohistiocytosis (HLH) [150]. The UNC13D mutation rs140184929 (c.2588G>A; p.[Gly863Asp]) is a missense variant that substitutes glycine with aspartic acid at position 863 (G863D), potentially impacting the protein’s functionality, especially regarding immune cell granule release [107]. Mutations in UNC13D have been associated with severe cytokine storms in patients with COVID-19, which may result in impaired immunological responses, thereby aggravating inflammation and increasing the risk of cardiovascular incidents during COVID-19 infection [107,150]. 

The DES gene encodes the desmin protein, which is an intermediate filament protein essential for preserving the structural integrity of muscle cells, especially in cardiac and skeletal muscle, where it significantly contributes to the cytoskeletal architecture and serves a role in muscle contraction and signaling [151]. The SPEG gene is essential for the formation, maintenance, and functionality of cardiac and skeletal muscles, with its expression reportedly elevated in the skeletal muscle, heart atrial appendage, and left ventricle in women compared with men [152]. Also, SPEG is a protein essential to muscle growth and functionality that is believed to contribute to the control of calcium signaling in myocytes, which is crucial for muscular contraction [153]. Regarding genetic predisposition to CVD, mutations in the DES and SPEG genes are associated with an elevated risk of cardiomyopathy, particularly the SNP rs71040457 [151,154]. This mutation is situated between the two genes on Chromosome 2 (chr2:219430060-219430061), downstream of the DES gene (3322 bp away) and upstream of the SPEG gene (4917 bp away), in which mutations in DES and SPEG may intensify cardiovascular complications in COVID-19 patients, resulting in increased death rates [155].

The LZTFL1 gene participates in several biological processes, such as cell differentiation and the control of immunological responses; it can be found in the epithelium of the normal lung and is involved in the transport of proteins to the cilia of the respiratory epithelial cells [156]. The SNP rs10490770 (chr3:45823240, T>C) in the LZTFL1 gene has been recognized as having a direct causative link with the severity of both COVID-19 and CHD [145].

The ABO gene encodes the glycosyltransferases that define blood group antigens, which are essential for blood type determination and participate in several physiological functions, namely immune system reactions as well as cell signaling [157]. Certain ABO blood types may be linked to variable chances of COVID-19 infection severity. It was shown that the ABO protein is a causative risk factor for severe COVID-19 symptoms and predisposition to COVID-19 [158]. The two ABO variants rs579459 (chr9:133278724, C>T) and rs495828 (chr9:133279294, T>G) have been investigated and shown to influence immunological response, potentially impacting the severity of COVID-19 and ensuing cardiovascular problems [145]. 

The gene known as ILRUN is responsible for encoding a protein that has a role in several processes, including lipid metabolism and the innate immune process signaling pathways [159]. The molecular features of ILRUN were shown to be associated with several diseases and disorders, including cancer, coronary artery disease, obesity, and viral infections. The ILRUN gene SNP rs2744961 (chr6:34687223, C>T) is purportedly linked to the severity of COVID-19 symptoms and cardiovascular events, possibly affecting the inflammatory reactions as well as the cytokine storm seen in severe instances, which indicates a common genetic route that predisposes patients to negative cardiovascular outcomes following COVID-19 infection [145].

**Figure 5:**
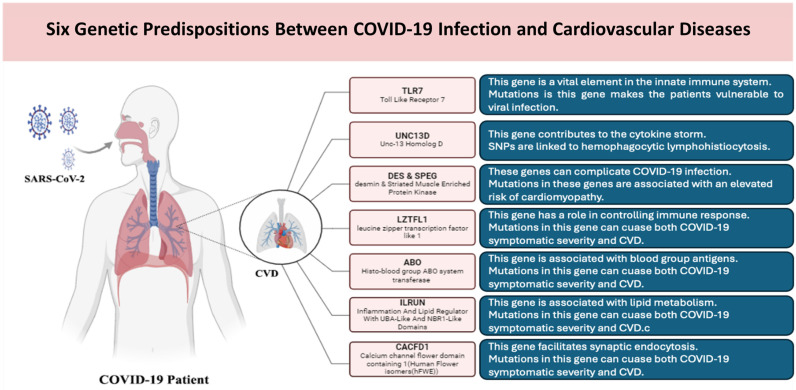
The six genetic predispositions between COVID-19 infection and cardiovascular disease (CVD). The figure highlights specific genes such as TLR7, UNC13D, DES and SPEG, LZTFL1, ABO, ILRUN, and CACFD1, detailing how mutations in these genes increase susceptibility to severe COVID-19 symptoms and related cardiovascular complications. Genetic variations in these genes contribute to immune dysregulation, cytokine storms, hypercoagulability, and inflammation, all of which elevate the risk of developing conditions like cardiomyopathy, heart failure, and thrombosis in COVID-19 patients.

hFWE1, hFWE2, hFWE3, and hFWE4 are the transmembrane proteins that are encoded by the CACFD1 gene. These proteins are also known as Human Flower isomers, and they are responsible for facilitating synaptic endocytosis and fitness-dependent cell elimination [160]. There are two SNPs in the CACFD1 gene that have been identified as factors that contribute to the genetic susceptibility for both COVID-19 and cardiovascular disease. These SNPs are rs4962153 (chr9:133458632, A>G/A>T) and rs3094379 (chr9:133469788, T>A/T>C). These two SNPs may have an effect on the underlying pathways that connect both cardiovascular events as well as COVID-19 symptoms severity [131]. How COVID-19 can cause CVD is represented in Figure 5.

When it comes to patients diagnosed with COVID-19, the complexity of the illness is readily evident, affecting various organs and systems, including the respiratory infection as the starting point. There are several variables that might impact the severity of disease symptoms. These factors include interindividual differences in age, sex, and comorbidities; behavioral factors such as eating, drinking, smoking, and stress; or genetic variants that influence physiological and pathological processes. Examples that are particularly noteworthy include genetic variations that influence the immune response pathways and cardiovascular functioning processes. These variants have the potential to contribute to the intensity of symptoms experienced by patients at increased risk as well as the occurrence of cardiovascular events. The results of these genetic factors in the genes (TLR7, UNC13D, DES, SPEG, LZTFL1, ABO, ILRUN, and CACFD1) provide substantial insights into the genesis of cardiovascular issues linked with COVID-19. These findings may also potentially guide personalized early diagnosis of disease and possible complications. In addition, the results may be of use in making educated medical decisions based on pharmacogenomic investigations, which are aimed at enhancing the effectiveness of pharmacotherapeutic options while simultaneously minimizing the risk of unwanted adverse drug reactions. Figure 5 illustrates six genetic predispositions between COVID-19 infection and cardiovascular diseases.

## 11. Future Research Directions

Heart failure and myocarditis are two common diseases that are brought on by viral infections, which have a major negative influence on cardiovascular health. Many questions persist despite progress in our knowledge of these interactions. Utilizing genome-wide association studies (GWASs) and epigenome-wide association studies (EWASs), future research must find new genetic and epigenetic markers associated with cardiovascular diseases generated by viruses. 

### 11.1. Genome-Wide Association Studies (GWASs)

A GWAS compares the genomes of people who are afflicted and those who are not to find genetic variations linked to diseases. Single nucleotide polymorphisms (SNPs) and other genetic variants that increase the risk of disease are revealed by these investigations. GWASs can detect genetic predispositions influencing an individual’s response to viral infections and the development of cardiovascular problems in virally induced cardiovascular disease [161,162]. For example, genetic variations in the ACE2 gene were linked to severe COVID-19 results in GWAS research. One example of how a GWAS might identify genetic markers influencing illness severity and susceptibility is the ACE2 gene, which is essential for viral entrance and replication [163].

Genetic loci linked to cardiovascular disorders like heart failure and coronary artery disease have been found by GWASs. There are, however, few studies that concentrate only on cardiovascular disorders brought on by viruses. Many studies have examined how genetic variables affect the risk of cardiovascular disease in general without taking viral infection effects into account [164]. Variants in the PCSK9 gene were linked to coronary artery disease and cholesterol levels by GWAS. Although this study contributes to our understanding of the hereditary risk factors for cardiovascular disease, more research is required to address viral-induced diseases [165].

A study used Mendelian randomization with GWAS data to identify COVID-19 susceptibility (β = 2.188 × 10^−3^, *p* = 0.002) and hospitalization (β = 8.626 × 10^−4^, *p* = 0.010) as causal risk factors for cardiovascular disease (CVD) death. Sensitivity analyses confirmed these findings with no evidence of bias or reverse causality. Genetic loci like rs505922 and rs2277732 were associated with COVID-19 severity and CVD risk, suggesting genetic and biological mechanisms including myocardial injury and ACE2-mediated damage. While robust, the study calls for further clinical research to validate these findings and improve COVID-19 management strategies [166].

### 11.2. Epigenome-Wide Association Studies (EWASs)

Epigenetic alterations linked to illnesses, such as DNA methylation and histone modifications, are identified by EWASs. These investigations look at how epigenome modifications affect gene expression and contribute to the pathophysiology of disease [94,167]. For example, an EWAS on heart failure found DNA methylation patterns linked to the severity of the condition [104]. These findings highlight how epigenetic modifications are linked to cardiovascular conditions and suggest that similar changes might occur due to viral infections. Additionally, an EWAS investigating DNA methylation in patients with COVID-19 myocarditis identified epigenetic markers linked to disease progression [168]. In viral-induced cardiovascular disease, EWASs can uncover epigenetic alterations induced by viral infections. 

### 11.3. Integrating GWASs and EWASs

**Figure 6:**
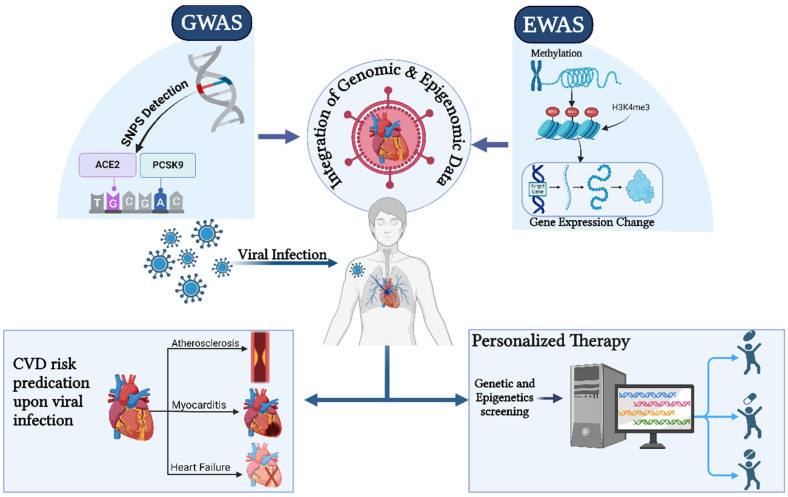
The integration of genomic and epigenomic data utilizing genome-wide association studies (GWASs) and epigenome-wide assoiation studies (EWASs) to identify cardiovascular disease (CVD) risk factors upon viral infection. GWASs detect genetic variants like SNPs in genes such as ACE2 and PCSK9, while EWASs focus on epigenetic changes, like DNA methylation, influencing gene expression. The combination of these data enables personalized therapies by screening genetic and epigenetic profiles, aiming to predict and mitigate CVD risks such as atherosclerosis, myocarditis, and heart failure in the context of viral infections.

A thorough understanding of how genetic and epigenetic variables interact to affect virally induced cardiovascular disease is made possible by integrating GWASs and EWASs [169]. Integrating information from the two types of studies can show how genetic variations and epigenetic changes interact to influence the development of and susceptibility to disease [170] (Figure 6).

It may be possible to determine how these variables interact by combining GWAS data on genetic variants linked to virally induced cardiovascular disease with EWAS data on epigenetic alterations [152]. The importance of integrating genomic and epigenomic data has been shown by recent studies. For instance, in autoimmune illnesses, combined GWAS and EWAS techniques have revealed genetic and epigenetic variables that influence the risk of disease. When applying comparable techniques to cardiovascular illnesses caused by viruses, new discoveries and treatment targets may become apparent [171].

### 11.4. The Need for Integrated Approaches

Cardiovascular illness involves complicated layers of gene regulation, protein interactions, epigenetic modifications, and metabolic changes that are not fully represented by investigating the genome alone. This is especially true in the setting of viral infections [172]. In order to address this complexity and achieve a more comprehensive understanding of disease causes, it is imperative to integrate many layers of biological data, including transcriptomics, proteomics, genomics, and epigenomics [173].

There are various benefits to integrating transcriptomic, proteomic, epigenomic, and genomic data in order to better understand the role that viral infections have in cardiovascular disease [174]. Combining the various omics layers can shed light on how modifications at one level impact processes at a later stage. Each omics layer reflects a distinct component of biological processes [175]. For example, genetic variants identified in a GWAS may influence disease risk by altering epigenetic marks, which, in turn, affect gene expression and protein function [176]. The way tissues and organs react to stressors including viral infections is ultimately determined by this series of biochemical alterations, which can result in conditions like inflammation, fibrosis, or even heart failure.

It has been demonstrated that viral infections such as those brought on by influenza or coronaviruses worsen cardiovascular diseases by inducing oxidative stress, inflammatory reactions, and direct damage to cardiac tissues [30]. But not everyone infected by the same virus experiences serious cardiovascular problems, indicating that host genetic and epigenetic variables are important in determining the likelihood and course of the disease. Utilizing multi-omics techniques, it is possible to decipher these intricate relationships between hosts and pathogens by connecting genetic inclinations to functional alterations in gene expression, protein synthesis, and cellular reactions [145].

Viral replication, pathogenicity, and host tissue tropism are complicated processes that have been clarified by combining genomic, transcriptomic, proteomic, and metabolomic data from the virus and the host. By focusing on important host components or pathways, this knowledge has helped develop antiviral treatments and repurpose already-approved medications [177]. Although little research has used multi-omics profiles to investigate processes related to cardiovascular diseases, this method has revealed the possible function of previously found GWAS loci and the mechanisms involved in these common disorders [175]. 

For instance, the area of the gene cluster CELSR2-PSRC1-MYBPHL-SORT at the 1p13.3 locus is linked to cardiovascular risk and low-density lipoprotein cholesterol (LDL-C) levels [178]. The ABO, NAT2, CPS1, NAT8, ALPL, and KLKB1 genes were among the loci identified in a study, [179], which conducted a GWAS for metabolite levels being linked to both metabolites and a high risk of CAD. Remarkably, bradykinin levels and an increased risk of CAD were linked to KLKB1. Bradykinin is a powerful endothelium-dependent vasodilator that is known to cause hypotension and vasodilation [180]. 

Although single-cell technologies have significantly improved the study of cell heterogeneity, several drawbacks still exist [181]. Accuracy, scalability, and sensitivity continue to be major obstacles that need to be overcome for ongoing technological advancements and complementing strategies. Dissociating cells is the foundation of many single-cell techniques, which limits spatial analysis and disturbs tissue architecture. This problem is addressed by spatial transcriptomics (ST); however, it has a limited resolution and frequently records signals from several cells, which could contaminate the data [182,183]. Furthermore, the transcriptome and proteome may change during the preparation of single-cell suspensions, which could compromise the accuracy of the data. Because bioinformatics methods differ in accuracy and parameters, which affect categorization and cell type identification, data interpretation is complicated [184].

Multi-omics research is further complicated by ethical and legal issues, such as patient privacy, data sharing laws, and ethical standards compliance [185]. These sophisticated approaches are available only to well-funded institutions due to the high expenses of data collection, sample processing, and specialized equipment, which further restrict accessibility and large-scale deployment [184].

### 11.5. Bridging the Gap Between Virology and Cardiovascular Research

Research on the relationship between viral infections and cardiovascular disease is essential because of the implications for personalized therapy and public health. It is becoming more widely known that viral infections, especially those brought on by pathogens like influenza and coronaviruses, can make cardiovascular disorders worse. This connection emphasizes the value of multidisciplinary research that unites the fields of virology and cardiovascular medicine and shows how customized medication can help reduce the dangers of viral infections on the cardiovascular system.

### 11.6. The Intersection of Viral Infections and Cardiovascular Disease

Through several ways, viral infections can have a major negative impact on cardiovascular health. The induction of inflammatory reactions is one main route. It has been demonstrated that viruses like the one that causes COVID-19, SARS-CoV-2, can cause systemic inflammation, which can worsen pre-existing cardiovascular diseases or result in new cardiovascular problems [1]. For example, patients with severe COVID-19 have been shown to have higher levels of pro-inflammatory cytokines and markers of systemic inflammation, which are associated with poor cardiovascular outcomes [1].

Furthermore, oxidative stress brought on by viral infections might harm cardiovascular tissues. Reactive oxygen species (ROS) generation and the body’s antioxidant defenses are out of balance, which leads to oxidative stress. The advancement of atherosclerosis and endothelial dysfunction—two major contributors to cardiovascular disease—are brought on by this imbalance [186,187]. Increased oxidative stress and ensuing cardiovascular problems have also been connected to influenza virus infections, underscoring the necessity of learning more about these processes [186].

The direct harm that viruses inflict on heart tissues is another crucial factor. Research has demonstrated that both acute and long-term cardiovascular problems can result from viral myocarditis. It has been shown that viruses like SARS-CoV-2 can directly infiltrate cardiac cells, leading to myocarditis and pericarditis that can seriously compromise heart function [21].

### 11.7. The Need for Interdisciplinary Research

For a thorough understanding of how viral infections affect cardiovascular health, it is imperative to close the knowledge gap between virology and cardiovascular research. An interdisciplinary study combined knowledge from the two domains to investigate the mechanisms behind this link and to find possible targets for treatment [188]. By merging knowledge from immunology, virology, cardiology, and molecular biology, collaborative efforts can result in a more comprehensive approach to the study of viral infections and cardiovascular disease [189].

Collaborating among experts from several domains, interdisciplinary study aims to uncover the processes behind the viral effects on the cardiovascular system. Virologists provide a profound comprehension of immune evasion tactics, viral activity, and the particular mechanisms by which viruses infect and harm host tissues [190]. Conversely, researchers studying cardiovascular disease shed light on how these viral interactions affect heart health, vascular integrity, and blood flow control. Immunologists contribute to our understanding of the immune response, especially the delicate balance between excessive inflammation and protective immunity, which can have detrimental effects on the cardiovascular system [144,191].

Furthermore, multidisciplinary partnerships facilitate the creation of novel diagnostic instruments. Through the integration of clinical cardiology, molecular biology, and bioinformatics, researchers can create biomarkers that predict cardiovascular risk in patients with viral infections [192]. By potentially identifying patients who are more prone to experience serious cardiovascular consequences before symptoms appear, these biomarkers can aid in early identification and prevention initiatives. For instance, improvements in multi-omics technology have made it possible to identify genetic and epigenetic markers that, in response to viral infections, suggest an increased sensitivity to cardiovascular disease [193].

Overall, studying viral infections and cardiovascular disease is generally made more comprehensive and integrated by interdisciplinary collaboration. Through the integration of knowledge from diverse domains, scientists can progress from discrete findings to all-encompassing approaches that tackle viral pathogenesis and cardiovascular consequences. The future of personalized medicine depends on this synergy since it will make it possible to create customized interventions that can more successfully lower the cardiovascular risks brought on by viral infections.

## 12. Challenges and Limitations of Multi-Omics 

The development of high-throughput data collection techniques at the gene network level has significantly advanced the profiling of transcriptomic signatures in human heart failure, offering new opportunities to explore cardiovascular diseases (CVDs), including those linked to viral infections [194]. However, while these technologies, such as genome-wide association studies (GWASs) and epigenome-wide association studies (EWASs), show great promise in uncovering disease mechanisms, they face several limitations and challenges that hinder their broad clinical application, especially in the context of viral infection-related cardiovascular diseases [195].

One of the primary challenges of using multi-omics technologies like GWAS and EWAS is the complexity of the data they generate, which require robust statistical methodologies and careful study design [196]. These datasets often present issues such as population structure variations, sample ascertainment biases, batch effects, and inaccuracies across different “omics” layers or technologies. For instance, a study integrating genomics with proteomics revealed that 25% of proteins had spurious protein quantitative trait loci (QTL) arising from coding variants overlapping with the epitope binding sites of the proteomics platforms used [197]. Such technical issues can introduce errors in the interpretation of the results and complicate the identification of true associations between genetic variants and cardiovascular diseases linked to viral infections [198]. 

Additionally, omics data are inherently high-dimensional, and researchers must contend with the heterogeneity of feature spaces across different datasets. Dimensionality reduction techniques or low-dimensional representations can mitigate these challenges, but the development of integrative tools for both unsupervised and supervised analyses is still ongoing [198,199]. These technical hurdles, alongside the challenge of interpreting vast numbers of associations, complicate the identification of biologically meaningful insights, particularly when the underlying biology of viral infection-related cardiovascular diseases is not fully understood.

Another significant limitation is the generalizability of discoveries made using GWASs and EWASs. These studies have faced criticism for their lack of population diversity, which is critical when assessing diseases such as viral infection-related CVDs, as genetic risk factors may differ across populations [200,201]. For example, a GWAS of coronary artery disease identified ancestry-specific genetic risk loci, underscoring the importance of considering population diversity in omics studies [202]. Furthermore, viral infections may influence CVD progression differently across diverse populations, and without broader representation, these studies may fail to capture important population-specific insights [203]. 

Similarly, the influence of sex differences in disease susceptibility and progression is often overlooked, even though many diseases, including cardiovascular conditions, exhibit sex-specific variations [204]. For instance, a study on atherosclerotic plaques found that male patients expressed immune and inflammation-related genes, whereas female patients had higher expressions of genes related to phenotype switching in smooth muscle cells. These sex-specific differences must be considered to fully understand the impact of viral infections on cardiovascular health [205].

Additionally, the reliance on tissue-based bulk transcriptomics analyses presents further challenges. Most omics studies use intact myocardial tissue samples, which include a mixture of various cell types and extracellular matrix contents, making it difficult to pinpoint the cellular origins of differentially expressed transcriptomic signatures [194]. Recent advances in single-cell RNA sequencing (scRNA-seq) and single-nucleus RNA sequencing (snRNA-seq) have provided powerful tools to overcome this limitation by allowing for more precise analysis of individual cell types. For instance, scRNA-seq has been used to map the transcriptional signatures of the normal human heart, helping to identify cell-type-specific gene expression patterns across different heart regions [206]. 

This approach also aids in understanding cell–cell communication, which is crucial when examining the effects of viral infections on the heart [207]. However, despite these advances, there remains a challenge in distinguishing the origins of different transcriptomic signatures, particularly when dealing with mixed tissue samples, which can limit the interpretability of the data in the context of viral infection-induced CVDs [208].

In conclusion, while GWAS, EWAS, and other omics technologies hold immense potential in improving our understanding of cardiovascular diseases, including those linked to viral infections, significant challenges remain. These include issues with data heterogeneity, population diversity, technical limitations, and the integration of multi-omics data. Overcoming these challenges will require the development of more robust statistical tools, better study designs, and broader, more diverse data sets to ensure that insights from omics research can be effectively translated into clinical applications, particularly for diseases like viral infection-related cardiovascular conditions.

## 13. Conclusions

COVID-19 has unveiled a profound link between viral infections and cardiovascular disease, underscoring the significant cardiovascular complications that can arise from SARS-CoV-2 infections. The interactions between direct viral invasion, immune dysregulation, and endothelial damage have provided new insights into the mechanisms behind viral-induced cardiovascular injury. The long-term cardiovascular consequences, including myocarditis, heart failure, and thromboembolic events, highlight the need for ongoing monitoring of recovered patients. Future research should focus on identifying genetic and epigenetic markers to predict cardiovascular risks in patients with viral infections. Integrating genome-wide and epigenome-wide studies will enable a better understanding of how genetic predispositions and viral infections interact, potentially guiding personalized therapeutic strategies. Moreover, interdisciplinary collaboration between virology, cardiology, and genomics is essential to address the complex mechanisms driving these cardiovascular complications. Personalized medicine, incorporating genetic and epigenetic profiling, can play a pivotal role in mitigating long-term cardiovascular risks in viral infection survivors, shaping a future where tailored interventions reduce the impact of viral diseases on heart health.

## Data Availability

All data are presented in the current manuscript.
